# “I thought if my parents got involved, then they'd make me get better”: emerging adults’ experiences of support from family and friends during anorexia nervosa

**DOI:** 10.1186/s40337-025-01260-8

**Published:** 2025-05-12

**Authors:** Rachel Potterton, Gary Brown, Ulrike Schmidt

**Affiliations:** 1 Department of Psychology, Royal Holloway, University of London, Egham, Surrey, TW20 0EX UK; 2 Centre for Research in Eating and Weight Disorders, Institute of Psychiatry, Psychology & Neuroscience, King’s College London, London, SE5 8AF UK

**Keywords:** Emerging adulthood; IPA; eating disorders, Anorexia nervosa, Family involvement, Parents

## Abstract

**Background:**

Around half of all eating disorder cases start during emerging adulthood (i.e., 18–25 years of age). This is an important time of change in interpersonal relationships, marked by individuation from the family of origin. Interpersonal relationships have long featured in theories of eating disorder maintenance and recovery. Increased understanding of the interplay between eating disorders and changes in the interpersonal domain may be key to improving the efficacy of existing treatments and developing novel interventions for this population group.

**Objective:**

This study aimed to explore experiences of support from family and friends amongst emerging adults with anorexia nervosa.

**Methods:**

A convenience sample of emerging adults who had received specialist treatment for anorexia nervosa in the United Kingdom (*N* = 10) was recruited via advertisements on social media. Semi-structured interviews were conducted focusing on experiences of support from family and friends during their eating disorder. Data were analysed using Interpretative Phenomenological Analysis.

**Results:**

Five key themes in participants’ experiences were identified: (i) feeling isolated and lacking close friends; (ii) resisting involvement of family due to perceiving them as part of the problem; (iii) feeling family and friends’ feelings; (iv) desiring flexible boundaries, and (v) feeling ambivalent towards family and friends’ lived experience.

**Conclusions:**

Findings suggest a complex entanglement of development of and recovery from AN with the process of individuating from parents during emerging adulthood. Clinicians may find benefit in helping emerging adults to develop their independence and supporting parents to adopt helpful emotional and behavioural postures that tackle the AN maintenance cycle, for example developing parental emotion regulation skills and supporting parents to facilitate age-appropriate levels of independence and responsibility.

## Abstract (plain language summary)

Eating disorders are common in young people aged 18 to 25 years old. This is also a time young people start to rely more on friends and partners for support, and less on family. We wanted to know what it was like when support changed in this way for people this age with anorexia nervosa. We spoke to ten such people and recorded what they said. We looked for themes in each interview. We matched these themes with similar themes in other peoples’ interviews.

These young people spoke about having few friends and relying on family for support. They told us they felt difficult family relationships was one of the reasons for having an eating disorder. It was important to them for family and friends to remain calm and control their own emotions so that young people could take the lead in their recovery. They told of mixed experiences when their family or friends had their own experience of eating disorders. Some thought this was helpful for their own recovery. Others said this was unhelpful. We hope these findings will help clinicians and policymakers to think about how they can help people with eating disorders get the most out of support from family and friends.

## Background

Emerging adulthood is the life-stage between adolescence and later adulthood (~ 18–25 years) [1, 2]. It is an important and exciting period of biopsychosocial development, whilst also being a time when as many as half the population meet criteria for mental health problems [3–6]. Eating disorders (EDs) - including anorexia nervosa (AN), bulimia nervosa (BN), binge-eating disorder (BED) and other specified feeding and eating disorder (OSFED) - are particularly prevalent during this life-stage [7–9]. EDs during emerging adulthood can have detrimental effects on ongoing development and long reaching and pervasive consequences, including negative impacts on educational and occupational productivity and overall quality of life [10, 11].

Despite the clear need for prompt and effective intervention for EDs during emerging adulthood, there are indications that emerging adults’ treatment needs are less well met than those of other age-groups with EDs [12–15]. Emerging adults tend to come to ED services with a longer duration of untreated ED than adolescents [12]. Others do not seek help at all, with up to 80% of emerging adults with clinically significant eating difficulties reporting not seeking help for these [7]. When treatment is received, emerging adults often report dissatisfaction with the care they receive [13–15]. It has been suggested that understanding EDs within the emerging adult developmental context may be key to addressing unmet treatment needs in this population group [16, 17]. Such understandings could be used to adapt existing treatments or develop novel interventions, thus improving clinical outcomes for this client group [16, 18–20].

There is a rich existing body of research which has explored the emerging adult developmental context extensively. Interpersonal relationships are well-established as a key focus of development during emerging adulthood. In sum, emerging adults gradually individuate from their parents and move towards committed and enduring romantic relationships and friendships as a source of emotional support [21–23]. Parenting behaviour is understood to play an important role in the individuation process [24, 25]. Studies have identified that emerging adults value low levels of control and encouragement of responsibility, in the context of continued warmth and support from parents [26, 27].

Relationships with family and friends have long featured in theories of the aetiology and maintenance of EDs. Psychoanalytic theorists have suggested that difficulties with separating from parents may be central to the aetiology of EDs [28, 29]. Whilst it is now accepted that EDs are complex disorders and cannot be attributed to any one single cause, it is also acknowledged that families can unintentionally maintain EDs [30–33]. Family-based treatment models, for instance, posit that parents may inadvertently accommodate the ED, for example by allowing their offspring to avoid social situations and engage in ED behaviours [31]. The cognitive-interpersonal model of AN, meanwhile, suggests that as families become worried about their offspring’s weight loss, they may become hostile or critical, or worried and concerned with a resultant focus on comfort and reassurance without any attempt to challenge the ED behaviours [33]. Conversely, family and friends can also be important sources of support; their encouragement appears to play an important role in treatment-seeking, and patients are often brought to treatment by concerned family and friends [34]. Supportive relationships are also viewed by many with lived experience as integral to recovery, as they provide a sense of feeling heard and understood and decrease feelings of isolation [35].

Interventions which endeavour to include family and friends in a developmentally appropriate way during emerging adulthood have started to come to fruition. These include the First Episode Rapid Early Intervention for Eating Disorders (FREED) service model, Young Adult Temperament-Based Treatment with Supports (YA-TBT-S), and family-based therapy for transition aged-youth [20, 36–38]. Results from evaluations of these interventions have been promising [20, 37–42]. Studies have shown that patients treated through the FREED model show greater clinical improvements than controls, and a qualitative study exploring patients’ experiences of the model found that participants felt being provided with opportunities to include family in treatment was deemed important to the success of the treatment [37–42]. Similarly, both YA-TBT-S and family-based therapy for transition-aged youth have been demonstrated to be associated with symptom reduction and weight gain [20, 36–38].

Further research is needed to understand how emerging adults with EDs use family and friend support during this life-stage. To the authors’ knowledge, just one qualitative study has focused specifically on exploring how emerging adults with EDs experience familial support [43]. In this study, participants perceived some parental involvement as helpful, but parents’ attempts to rigidly control or limit young adults’ autonomy were experienced as unhelpful. Whilst practical support was regarded as helpful, emotional support was the more significant and enduring mode of support and helped to reduce feelings of isolation. All participants in the study had transitioned from child and adolescent to adult ED services in the preceding two years; the study, therefore, captures the experience of emerging adults during transition between services. However, it is not clear what form parental support takes when the emerging adult has been in adult ED services for some years. It is also not clear what form support takes amongst those who seek help from adult services without any prior experience of child and adolescent services. Child and adolescent services tend to differ markedly from adult ED services with respect to how they understand the role of family and friends in the maintenance of and recovery from EDs [18, 44–45] In child and adolescent services, parents are considered integral to intervention efforts, and family-based treatment is the front-line treatment [31]. Meanwhile, in adult ED services, family and friends are considered much less integral to intervention, with a foregrounding of individual-focused psychological treatments (e.g., enhanced cognitive behaviour therapy for EDs, The Maudsley Model of Anorexia Nervosa Treatment for Adults [MANTRA] [33, 46]. This previous study also focused exclusively on familial support; it is important to explore further the role of loved ones (e.g., friends and romantic partners) other than family members. Finally, as the previous study was conducted in Canada - which has a different healthcare system to the United Kingdom (UK) - it is not clear the extent to which the findings are relevant in a UK context. Whilst research is needed to understand common factors across healthcare systems, it is also important to explore factors unique to different health systems and contexts.

Further research on emerging adults’ experiences of eating disorder related support from family and friends is therefore needed. This study therefore aims to extend the limited evidence in this area by exploring how treatment-seeking emerging adults with AN experience support from family and friends.

## Methods

### Design

The study used a qualitative design, collecting data via semi-structured interviews and analysing these data using interpretative phenomenological analysis (IPA) [47]. IPA is underpinned by three theoretical frameworks: phenomenology, idiography and hermeneutics. Phenomenological approaches suggest that human experience can be studied and understood through detailed examination of participants’ perceptions, without regard to predefined theoretical categories [47]. Idiography, meanwhile, means that IPA it interested in not only how participants’ experiences converge, but also how they differ. IPA therefore focuses on analysing single cases before making comparisons between cases. Lastly, IPA is concerned with hermeneutics, which acknowledges that humans are sense-making beings [47]. IPA is particularly concerned with the “double hermeneutic” intrinsic to attempts to understand another’s lived experiences, whereby any account of the participants’ sense making is also inevitably filtered through the researcher’s own sense-making [47]. IPA does not try to eliminate the researcher’s interpretative act but instead aims to contextualise findings within the researcher’s own worldview.

The study is reported according to the Consolidated Criteria for Reporting Qualitative Research Guidelines (CORE-Q) [48].

### Setting

The study was conducted in the UK between August 2023 and May 2024. A convenience sample of participants was recruited via advertisements posted on social media and via inclusion in a newsletter advertising current opportunities to participate in research, circulated via email to all students enrolled at King’s College London.

### Participants

Participants were eligible for inclusion in the study if they (1) had received treatment of any type (e.g., psychological, medical, dietetic) in a specialist ED service for an ED specified in the DSM-5; (2) this treatment took place in the UK; (3) treatment occurred whilst the participant was an emerging adult (i.e., 18–25 years of age); (4) treatment was completed between one and three years prior to participation in the study. An upper limit on time elapsed since treatment was selected to ensure detailed and accurate recall of experiences. A lower limit on time elapsed since treatment was selected as EDs– AN in particular - are often egosyntonic (i.e., positively valued by the client) [49]. Furthermore, malnutrition and related psychopathology during the acute phase of illness are likely to influence cognitive and reflective capabilities [50, 51]. It was thought that focusing on the meaning participants had made of family and friend support once the ED was less positively valued, and the participants were better positioned to engage in reflective thought, was more likely to generate constructive insights that could be used to inform intervention development.

Recruitment ceased when 12 participants were recruited. A sample size was not decided upon a priori. The decision to cease recruitment at this point was made considering the richness of the data collected, the desire to examine similarities and differences between participant accounts, and the pragmatic restrictions of the project [52]. Analysis was conducted alongside recruitment and data collection, allowing the researchers insight into the richness of the data collected and therefore guiding sample size decisions. The researcher did not have any professional or personal relationship with any of the participants prior to study commencement.

Following the analytic process, it was decided to exclude two participants from the study. This decision was taken as these two participants reported diagnoses of BED, whilst the other 10 participants reported AN. BED and AN are phenomenologically distinct illnesses, thought to be characterised by distinct underlying mechanisms [53]. Additionally, these two participants with BED happened to also be the only non-White individuals in the sample, and one was the only male participant. It also became clear during the analytic process that their data did not align with the predominant themes emerging from the data. It was therefore considered more methodologically robust to exclude these participants and focus solely on the participants with AN, thereby ensuring a theoretically coherent sample.

Participant characteristics are summarised in Table 1. Participants were all female, White British, and had received treatment for AN. Half of the participants had received treatment in child and adolescent ED services as well as adult ED services, whilst half had received ED treatment for the first time in adulthood. 70% of participants (7/10) had experience of inpatient ED treatment.

**Table 1:** Participant characteristics

Participant Number	Pseudonym	Age at Interview	Gender	Ethnicity	Self-reported Diagnosis	Transition from CAEDS	Inpatient Treatment	Interview Duration in Minutes
1	Jane	30	Female	White British	AN	Yes	No	53
2	Megan	25	Female	White British	AN	Yes	Yes	56
3	Jess	35	Female	White British	AN	No	Yes	60
5	Emily	21	Female	White Other	AN	Yes	No	58
6	Isabelle	23	Female	White British	AN	No	Yes	58
7	Sienna	24	Female	White British	AN	Yes	Yes	57
8	Elizabeth	23	Female	White Other	AN	No	No	49
9	Blanca	21	Female	White British	AN	Yes	Yes	42
11	Daisy	27	Female	White British	AN	No	Yes	37
12	Lucy	25	Female	White British	AN	No	Yes	47

### Reflexivity

The first author attempted to bring to consciousness the experiences relevant to their sense-making at several points during the data collection and analytic process. The first author wrote memos of personal responses to the data collected during interviews and noted personal responses to the data during the exploratory note-taking phase of analysis. In the interests of contextualising the study’s analysis and findings, it is highlighted here that at the time of data collection and analysis, the first author was in their early 30s, and so had relatively recent first-hand experience of emerging adulthood. The first author was also a trainee clinical psychologist with experience of both conducting research and practicing clinically in the ED field.

### Procedure

Interested parties were invited to contact the first author via email. A participant information sheet was shared, and eligibility was confirmed. A mutually convenient time and date for the interview was then arranged. Interviews were conducted on a one-to-one basis by the first author using a secure online video platform (Microsoft Teams). Following a consent process, participants were asked questions about selected demographic characteristics (e.g., ethnicity, gender, age), before proceeding with the main interview. Interviews were structured around a topic guide developed by the authors and piloted with three experts by experience. Engagement with existing literature was minimised during topic guide development. Five broad questions were devised, focusing on exploring participants’ experiences of support from family and friends. The topic guide was used flexibly and conversationally [47]. Data collection and analysis were iterative processes, with some areas of questioning excluded and new emerging areas of questioning included in the topic guide as data collection progressed.

On interview completion, participants were provided with a debriefing sheet. Participants were also emailed a £10 voucher to compensate them for their time. The interviews ranged from 37 to 60 min, with the average length being 51 min. All interview audio was recorded using the recording function in-built to Microsoft Teams. This function also provides a transcript, which was checked by the first author against the audio recording for accuracy and corrections made as needed. All identifying information was removed at point of transcript checking, and participants were assigned pseudonyms. No repeat interviews were carried out, and transcripts were not returned to participants for comment and / or correction due to time and budget constraints associated with the project.

All data analysis was solely conducted by the first author, without the use of assistive software. Analysis adhered to the detailed description of the IPA process by its creators [47]. In line with the IPA process, themes were not identified in advance of analysis and a coding tree was not used. As per IPA’s idiographic focus, analysis was conducted for each participant individually to begin with. The researcher read each transcript, making exploratory notes of initial reactions in the margins of each participant’s account. Exploratory notes were influenced by the theoretical underpinnings of hermeneutics and phenomenology, i.e., focused on understanding experience and both the participants’ and first author’s own sense-making. Following completion of the noting stage, experiential statements succinctly capturing portions of the text were formulated. Once experiential statements were identified for the whole interview, these were clustered together. Once the clustering felt satisfactory and meaningful, each cluster was named as a personal experiential theme, where each theme was an expression of the convergence of the experiential statements. An individual table of personal experiential themes was then constructed for each case. These tables were printed out, and cross-case analysis conducted whereby high-level similarities were identified alongside idiosyncratic differences in experiences at a more specific level. The analytic process continued into writing up, with group experiential themes continuing to be refined in discussion with the other authors. Participants did not provide feedback on the findings due to time and budget constraints associated with the project.

## Results

Five superordinate experiential themes were identified. These themes and associated subordinate themes are presented in Table 2.

**Table 2:** Experiential themes

Theme Number	Experiential Theme Name	Experiential Subtheme Name
1	Feeling isolated and lacking close friends	
2	Resisting involvement of family due to perceiving them as part of the problem	Wielding the ED as a way to feel in control when feeling overprotected
		Using the ED to elicit care when feeling deprived
3	Feeling family and friends’ feelings	Utilising the ED to manage emotional contagion within the family
		Longing for acknowledgment from family
		Regulating feelings with the help of family
4	Desiring flexible boundaries	Resisting increased control by family
		Longing for boundaries to be set
		Others allowing space
5	Feeling ambivalent towards family and friends’ lived experience	

### Feeling isolated and lacking close friends

Isabelle, Jess, Daisy, Blanca, and Megan all described feeling socially isolated, and having few close friends when they were experiencing AN. Isabelle, for instance, reflected that *“my two closest friends were living away at uni [when I had AN]*,* so they were far away physically.”* Jess, meanwhile, said that *“I went to uni and I didn’t really keep up with anyone from home.”* In both Isabelle and Jess’ accounts, AN was experienced at a time of change in their friendships, as a result of the transition to university. Common to both accounts is a description of geographical distance between them and their existing friendship groups. However, the effect of this distance on intimacy differs - whilst Isabelle’s account suggests that emotional closeness remained despite the changed geography, Jess’ account highlights how physical distance engendered decreased intimacy with friends. Blanca’s account, meanwhile, offered a sense of her keeping her peers who had physical proximity (i.e., new flatmates) at an emotional distance, and avoiding the development of new bonds to replace those that had been lost during the transition to university. She reflected that *“[my flatmates and I] didn’t really talk […] I think it was probably the best for me because it did just mean I can go into the kitchen if I am going to eat*,* you know*,* an entire plate of mushrooms*,* no one’s going to look at me and go - that’s kind of weird.”* In this extract, Blanca understood this emotional distance as being in her own best interests, because it allowed her unusual eating habits to go unquestioned. Isabelle, Jess, Daisy, Blanca, and Megan therefore all felt the absence of supportive friends, although the extent to which participants saw themselves as active participants in this isolation differed. For some, it was an inevitable consequence of the life-stage, whilst for others it was an intentional choice motivated by a desire to maintain anorexic behaviours.

### Resisting involvement of family due to perceiving them as part of the problem

Participants expressed that relationships within the family– and between them and their parents specifically– had contributed to the development of their eating difficulties. Within this theme, there were two experiences highlighted by participants: wielding AN to feel in control when feeling overprotected and, secondly, using AN to elicit care when feeling deprived of emotional support and care.

**Wielding the eating disorder to feel in control when feeling overprotected.** In Sienna, Megan, Blanca, Jane, and Lucy’s accounts, they experienced parental figures as overprotective and allowing them very limited independence when they were growing up. Megan, for instance, reflected that *“my granddad’s always been very controlling with me and sort of like…. control where I go*,* when I go and […] like I wanted to go into medicine*,* and he was like*,* no*,* it’s too hard for you.”* Here, the constraints her grandfather placed on her in relation to her career options were understood as coming from a desire to protect her. In Sienna’s account too, there was an acknowledgement of parental good intentions underpinning overprotection: *“I think I’ve definitely always not had much from [my parents] independence wise. I mean*,* it all comes from the right place.”* Both Megan and Sienna described feeling increasing dissatisfaction and frustration with their parents’ perceived overprotectiveness as they got older, craving distance from their parents. Megan shared: *“I think I found that hard*,* that I have no control over*,* sort of what was… going on in my life.”* Parental overprotection was therefore experienced as well-intentioned, but increasingly problematic as they aged into emerging adulthood and craved more independence.

In this context, Sienna, Blanca, and Jane experienced AN as a (sometimes inadvertent) route out of suffocating overprotection by parental figures. Sienna shared that *“I didn’t even know how to use a washing machine until I went into hospital [for AN]*,* and then like everyone else know exactly what to do. And I’m like*,* oh my God*,* I don’t know how to cook.”* Here, a hospital admission prompted by Sienna’s AN seemed to catapult her into adult life and its responsibilities and was associated with a sense of awakening to parental sheltering. This is less starkly depicted in Blanca and Jane’s accounts, but there is a sense of AN being a source of control and independence in the context of overprotection. For instance, Jane recalled that *“I suppose quite a lot of the reason why I sort of went into [AN] was stemmed around the control of [my parents]… over-controlling by them. So*,* they were always close*,* but with the distance because…. I needed my own control.”* Here, Jane seems to portray the AN as a conscious, considered response to parental overcontrol. Blanca, meanwhile, shared *“I was now aware of the fact that*,* yes*,* someone could say*,* you know*,* you must eat everything. But also*,* I know that they can’t do anything about that.”* In this account, engagement in restrictive eating was experienced as triggering an awareness of the limits of Blanca’s parents’ authority and control over her. Despite differences in the nuances of experience, there is a sense across these accounts of AN being associated with feelings of agency and increased independence from parents. For others, including Lucy and Sienna, parental authority was still feared, and increasingly restrictive eating was a rebellion staged in private, whilst the appearance of compliance with parental authority was maintained. As Lucy shares, “*It felt like I would lose a lot of control if I told [my parents about the AN]. I needed independence and I couldn’t see a way in which I tell them*,* and they wouldn’t want me to be living with them […]. I think there was an element of fear that they try and make me recover*,* I suppose.”* Similarly, Sienna reflected that *“I thought if [my parents] got involved then they’d make me get better.”* Here, anorexic eating patterns provide an internal feeling of agency and control, but their exposure is associated with the threat of increased parental control.

**Using the eating disorder to elicit care when feeling deprived.** Elizabeth and Isabelle described family backgrounds experienced as emotionally neglectful and invalidating. Elizabeth, for instance, reflected that: *“I think my parents have always been very closed off and detached”.* In this quotation, there is an understanding of this as an in-built and long-standing parental disposition, and an accompanying feeling of resignation. Isabelle, meanwhile, shared that: *“I think my younger sister was 13 and my brother was 12. It was quite like a busy house.”* In contrast with Elizabeth’s account, there is more a sense here that that feelings of neglect are situational rather than dispositional. Similarly, Jess describes a situational change in closeness to her parents, in her case prompted by increased physical distance between her and her family when she left the family home to attend university: *“so I went [to uni]… so it’s about a three-hour drive at that point and […] I was very homesick*,* I’ve always been quite a family person and I’ve never really spent much time away.”* Whilst the precipitants vary, there is a sense across these accounts of parents unable to meet the emotional needs of their emerging adult offspring.

In the context of feelings of emotional deprivation, Jess and Isabelle experienced the AN as inadvertently garnering care and attention from parental figures, allowing the parent to take over and them to be cared for in a way that a young child would be. As Jess recalls, *“I had a complete and utter breakdown […] I decided that I needed to go back home […] I moved in and lived with my auntie […] So she was kind of…. she then regulated my eating and kind of was…. what you need to do type thing.”* Similarly, Isabelle shares *“When I came out of the like general mental health unit*,* I moved into my mum’s house instead of my dad’s house [….] I did probably like regress*,* I was*,* like 20 years old but I think it was kind of like. You know*,* like when you’re*,* like*,* poorly*,* when you’re like a baby and like your mum looks after you*,* it was kind of like that.”* For both, AN prompted the return to the familial home, and a regression to an earlier stage of development, with a parental figure providing increased care.

### Feeling family and friends’ feelings

Participants shared experiences of feeling very aware of family and friends’ emotional responses to the AN. There were three subthemes in participants’ experiences: participants utilising AN to manage emotional contagion within the family, a longing for acknowledgment from their family, and experiences of regulating their feelings with the help of their family.

**Utilising the eating disorder to manage emotional contagion within the family.** Sienna, Daisy, Megan and Jane described feeling that family members were unable to regulate their own intense emotional responses to the AN. Sienna, for instance, shared that *“my mum would be crying all the time. I literally could hear her crying in bed and stuff.”* Megan described experiences of family members being unable to regulate their anger and frustration, lashing out with comments experienced as hostile or critical. For instance, Megan recalled that *“my granddad said I was making it up*,* said I was attention seeking… but my uncle said that I was just weird.”* Whilst the emotional tone of the parental outbursts in these accounts are different, there is a shared sense of the emotional pain they engender in their offspring.

The emotional burden of parental outbursts was further highlighted by Daisy and Megan, who described how their experiences of these overt displays of emotion elicited negative feelings such as guilt and frustration in them. Daisy recalled that *“I just felt so guilty because I wasn’t doing it to upset [my mum] and it frustrated me that she couldn’t see that I was doing it to feel better about stuff as opposed to*,* like*,* doing it to upset her”.* Megan shared similar feelings of frustration as a result of misinterpretation of her intentions: *“[my family would say] you’re doing it for attention kind of thing and… which wasn’t*,* what*,* I found it really difficult because it wasn’t that at all… it made [the AN behaviour] more likely because then I felt bad*,* that [my mum] was upset. So then I turned to the thing that at the time was my way of coping*,* to try and make myself feel better.”* Here, Megan highlights how anorexic behaviours are experienced as a way to cope with her emotions, and how emotional contagion within the family therefore has the unintended effect of worsening her AN.

**Longing for acknowledgment from family.** In contrast to the overt displays of distress characteristic of the previous theme, Elizabeth and Blanca described their experience of parents who appeared unreactive to the AN. Elizabeth, for instance, shared *“my parents have been very emotionally distant for all my life*,* and I think at this point*,* I was screaming for help. And still*,* they’re so nonchalant about it.”* In this extract, Elizabeth understood her AN as a communication– it is her *“screaming for help”.* There is a wish here that this communication will overcome a long-standing emotional distance and facilitate connection with her parents. However, they are *“nonchalant”*, a word which conveys a sense of them having heard her cries for help but seeming to be unperturbed by it. Meanwhile, in Blanca’s account, there were clues as to what acknowledgement was desired from family; she shared *“all I wanted was for someone to say I know that you are sick*,* you are unwell.”* There was a wish for AN to be understood as an illness, and for verbal acknowledgement of this. She went on to explain why this form of acknowledgement was important: *“I needed the validation of her being like OK*,* I know you’re unwell*,* because you’re unwell we need to do these things*,* rather than just being like oh*,* Blanca’s just eating lots of food and gaining weight for fun.”* AN had to be acknowledged as an illness because this gave permission for a move towards recovery– it legitimised increased food intake and consequent weight gain as a need, not a desire. In both accounts, lack of parental acknowledgement of difficulty makes movement towards recovery more difficult.

**Regulating feelings with the help of family**. Emily and Blanca described the importance of family members who were able to remain calm and regulate their own feelings. Emily in particular spoke of her mother’s ability to regulate her own emotions and remain calm: *“[my mum’s] quite calm and quite steady. Which was massively helpful*,* especially when like I couldn’t regulate at all. She was able to do that. […] It just felt like nothing was safe*,* nothing was stable in my life*,* and at least my mum was very*,* very consistent*,* and dependable. And like I very much knew I was loved*,* from my mum.”* Here, there is an implied entanglement of Emily and her mother’s ability to regulate; mum can regulate herself, which in turn helps Emily to do the same.

### Desiring flexible boundaries

Participants shared their experiences of family and friends’ behavioural responses to the ED, and their experience of longing for some boundaries to be set by others, but for these not to be overly rigid or obtrusive. There were three sub-themes in participants’ experiences - resisting increased control by their family, a longing for boundaries to be set, and others allowing space.

**Resisting increased control by family**. Blanca, Sienna and Elizabeth described experiencing their parents as responding to the ED in an authoritarian and controlling manner, for instance, by taking it upon themselves to monitor their weight or whereabouts. Blanca shared that *“[my mum] definitely very much likes to be involved in my weight […] sometimes she would whip out the scales and go. OK*,* step on. Which was the least helpful thing she could do.”* There is a conviction in this extract, conveyed by the *“definitely very much likes”*, that mum’s control is almost gleeful, rather than concerned. Elizabeth in contrast recognised in hindsight that their parents’ attempts to control were motivated by concern: *“at the time*,* I didn’t see it as…. they’re being supportive or caring. It just seemed like they’re being mean for the sake of it.”* Whilst there are idiosyncrasies in their experience of the intention behind this control, across both Elizabeth and Sienna’s accounts, there is a sense of parental efforts to control being counterproductive. As Elizabeth recounts, “*my immediate reaction [to my parents’ increased control] was… to pull away [from them]*,* to isolate myself and to stop eating. That’s just kind of how I deal with… and I don’t know why. It feels like a retaliation against somebody else…. If I don’t eat.”* Parental interference therefore had the opposite of the desired effect; it was construed as an attack that warranted retaliation and self-assertion, by further engagement in self-starvation. Similarly, Sienna shared *“when someone’s like pouncing on me. Like*,* what’s wrong? Why aren’t you eating this? Why aren’t you doing that then? […] I feel claustrophobic. I feel suffocated and I’d like to do things my own way […] It’s a control thing as well*,* because I want it to be in control of my recovery […] I couldn’t do it when someone was telling me to do it.”* The word *“pouncing”* here again suggests a physical attack by parents that necessitates escape and defiance. Across both accounts, there is a clear sense of parental attempts at control leading to an intensification of anorexic behaviours, which are construed as acts of self-determination and defiance.

**Longing for boundaries to be set**. Whilst ill, Isabelle and Jess experienced their mothers as trying to protect them from feeling distressed. Jess, for instance, described an overly prolonged trip to the supermarket where anorexic indecision is facilitated: *“[my mum] kind of gave in because she saw how upset I was. Like I remember going shopping and us being literally*,* we got a parking ticket because we were in the supermarket for so long… Because I couldn’t choose what to eat… she’s not forceful.”* There is disdain here for the lengths Jess’ mother went to to avoid distressing her; she chose to get a parking ticket rather than hurry Jess along. Jess went on to clearly express a wish for more rules and boundaries: “*There wasn’t enough challenge. It was all too enabling. It was too easy for the eating disorder to just walk all over [my mum]….my auntie was more bossy […]*,* I feel as if there’s less boundaries to be pushed with her.[…] My eating disorder hated being with my auntie*,* so more helpful in so far as I think it knew that it could always get around mum*,* there was always negotiation to be had*,* whereas with my auntie there was no negotiation.”* Here, Jess places distance between herself and the ED– it is externalised and portrayed as sneaky, looking for ways to *“get around”* people. In this context, her *“bossy”* aunt fares better than her mother and seems to be therefore regarded with more respect. Jess described friends having a similarly accommodating approach to her mother: *“they would say “you can come out with us*,* and you can not have anything to eat. That’s fine.”… Like “we have your back.” It’s difficult to know*,* isn’t it*,* whether that was helpful or whether it’s just enabling the eating disorder.”* In contrast to her certainty that her mother’s approach was unhelpful, Jess expressed more uncertainty about the helpfulness of her friends’ approach. There seems to be a desire for others to challenge the AN, alongside an underlying acknowledgment that it is harder for friends than family members to do so.

**Others Allowing Space.** Sienna, Blanca, Emily, and Jane described wanting to be given space, to have others step back and allow them to take the lead in their recovery. As Sienna shared, *“I think I just needed space [to move towards recovery]”.* For Blanca and Emily, leaving home and the transition to university was experienced as a key moment of change in the relationship with their parents, where parents had to relinquish control. As Blanca shared, *“I was like three or four hours away*,* there was physically nothing [my mum] could do. Like*,* she couldn’t force me to go to therapy. She couldn’t force me to do things. It was kind of… It’s up to you what you do.”* The choice of the words *“force”* and “*physically”* here suggest that with physical proximity comes a fear of being physically controlled, intruded upon, almost manhandled, by parents. This threat is neutralised by increased physical distance and comes with a sense of relief and increased freedom.

Both Sienna and Emily acknowledged that increased physical distance from parents did not uncomplicatedly foster movement towards recovery; As Sienna shared, *“I was really excited to get away from home and for a fresh start and I thought I’d be OK*,* but the back of my mind*,* I knew I could use this as a chance to just not eat.”* This extract speaks to a sense of inner conflict and ambivalence that increased freedom engenders. Similarly, Emily shared an increased engagement in anorexic behaviours in her first year of university, and an acknowledgement that *“if I was at home*,* I would not have become as unwell as I became in my first year of university”.* Both shared feelings of ambivalence about recovery and expressed how increased independence from parents was used in an opportunity to increase anorexic behaviours.

However, Sienna, Emily and Blanca described how increased physical distance from parents, and consequent increases in ED behaviours, helped them realise their own agency and learn that their actions have consequences. As Emily shared, *“I needed that period [of getting more unwell] to… understand…. the power I had over my life choices when I had nobody to push up against. I was the only one facing any consequences for that […]. I really had to look at my situation and decide - do I want to be […] a career anorexic or I could have a lot more control over my life and what I wanted.”* In this extract, Emily seemed to credit AN, unbridled by parental supervision, with increased feelings of power and agency. However, AN seems to be understood as a temporary experiment in self-determination, which should be discarded once this purpose has been achieved. If pursued for too long and too fervently (as a *“career”*), it itself becomes associated with a loss of control.

Crucially, whilst Emily and Blanca’s accounts emphasise the important of space from parents, they do not describe total freedom from parental oversight. When away at university, they remained in contact with parents and described some monitoring by parents. However, this was done in a much less obtrusive way than previously. As Blanca shared, *“[my mum] would just talk about like*,* oh*,* I went to Lidl this week and I bought this. Oh*,* have you bought anything interesting recently? Like*,* it was kind of a subtle […] it wasn’t “are you eating? I’m going to corner you until you answer me”.”* The tentativeness and unobtrusiveness of this probing contrasts with Blanca’s earlier imagery of being physically intruded on by her parents. Emily, meanwhile, also spoke to a transformation of support provided by her mother following her transition to university: *“knowing that [my mum] was there and was that safety net that if I was really unwell*,* she could have like*,* pulled the plug.”* The images of a safety net and a plug being pulled convey the idea of her mother having some ultimate oversight. There is a sense in both accounts of continuing to feel contained by parents.

Whilst for Emily, Sienna, and Blanca, moving away to university was a key catalyst for change in how support was provided by parents, for Jane, meeting a supportive partner was an important transition. Jane described the development of this new attachment relationship, and her partner replacing her parents as her main support. ***“****My parents were so involved when I was younger*,* and [my partner] sort of took over that role*,* but it was different because he left me to it a lot more than them […] a bit more laid-back really*,* I suppose…. Whereas if my mum saw me do something*,* she would be like*,* why are you doing that? What are you doing?”* Jane contrasts her partner’s responses to her mum’s; whilst mum is portrayed as anxiously surveilling from a position of authority, her partner’s support is experienced as much less obtrusive and more equitable. There are similarities here to Emily and Blanca’s parents’ support once they had moved to university– it is altogether calmer and less suffocating.

Jess offered another perspective on the theme of space; she reflected on the importance of her parents acknowledging the space between her and the ED: “*All [my mum and I] spoken about since I was 16 is my eating*,* and I’ve asked her specifically now to not ask me about my weight*,* my food. She actually said to me the other week*,* “but what am I meant to talk to you about then?” […]. I want to just have a normal mother-daughter relationship with her. I can’t because she isn’t able to…. detach me… from the eating disorder.”* In this extract, there is a sadness or wistfulness that Jess’ mother had not been able externalise the AN; it therefore takes up all the space in their relationship, and this is experienced as unhelpful.

In contrast, Jane, Isabelle, and Daisy commented on how friends had facilitated a sense of space and remove from the AN. Isabelle, for instance, reflected that *“to just hear [my friends] talk about their life reminds me that there’s life outside hospital*,* because I think the hospital is such a bubble.”* Similarly, Jane shared *“it was just nice to go out with [my friends] and be normal […] And feel like you can interreact….with*,* with everybody as normal and you’re not that far off from being like them.”* In these accounts, others offering space was again construed as important for recovery; friends facilitate engagement with aspects of identity outside of AN, which fosters hope and motivation for a life without AN.

### Feeling ambivalent towards family and friends’ lived experience

Many participants described having family or friends with lived experience of ED. Several participants described family and friends’ lived experience of EDs as detrimental to their recovery. Jess, Isabelle, and Blanca described their experience of having mothers with experience of EDs or disordered eating. Isabelle shared *“I’d go on home leave*,* and I’d have to have my breakfast and like dinner*,* tea*,* supper and two snacks a day. And then like in my head I’d be thinking [about mum]*,* “but you’re not eating enough today”.”* Similarly, Blanca shared that *“sometimes [my mum] would whip out the scales and go*,* OK*,* step on […] she would sometimes step on them after me and I would see her weight*,* which was less than mine*,* which was just useless.”* In both these accounts, there is a competitive element to the mother-daughter relationship, which is experienced as profoundly unhelpful.

In contrast, lived experience was understood by some as facilitating family and friends to provide better support. Sienna and Blanca described family members with lived experience being able to pick up that something was wrong, and seeming to intuit what support was needed, which made them feel understood. As Sienna shared, “my dad had anorexia when he was younger […] so he kind of gets it […] it felt like he knew how I was feeling, so he knew how to respond.” Blanca, meanwhile, shared *“It wasn’t something that I had to tell [my sister] to do. I think she just kind of knew [….]. She kind of had similar experiences*,* whereas obviously my mum hasn’t had those experiences.”* There is a sense here that lived experience negates the need for communication of experience; they are able to understand without ever needing to be told.

Both Sienna and Lucy described having made close friends in ED services. Lucy shared *“since being discharged Maisie and I have supported each other so much. Just because we both understand what each other’s going through.”* Meanwhile, Sienna explained *“I’ve got a really good friend that I met in day-patient […] it was nice to be able to relate to her. […] And I think that was really*,* really important in my recovery.”* For both Sienna and Lucy, the strength and value of these friendships in facilitating recovery seems to lie in a sense of shared experience and mutual understanding.

## Discussion

This study is one of the first to explore emerging adults’ experiences of family and friends’ support when experiencing AN. The study identified several key characteristics of the emerging adults’ experiences. Findings suggests a complex interplay between both the aetiology and maintenance of AN, and the process of individuating from parents during this life-stage.

This study found that emerging adults with AN experienced feeling isolated and lacking close friends, in part due to their desire to maintain their ED behaviour. This is a novel finding and was not touched upon by the previous study in this area, most probably because previous research focused exclusively on experiences of familial support [43]. The finding is consistent with other studies which have found that EDs are associated with difficulties in friendships and contribute to feelings of social isolation at university [60]. These findings contrast with the broader developmental literature, which has found that emerging adulthood is a time when, typically, young people start to rely more on friends and romantic partners as sources of support [17, 18, 54]. It seems that the ED may, for some emerging adults negatively impact normative individuation processes, and that this in turn contributes to ED maintenance.

This study’s findings also indicated that participants resisted involvement of family members due to perceiving them as intrinsic to the development of the AN. This is a novel finding and was not a key theme in the previous study in this area [43]. However, it does broadly align with existing research on what emerging adults require of parents during this life stage, namely, to relinquish control, in the context of continued emotional warmth and support [26, 27]. Findings are also consistent with existing theory and research which have suggested that EDs may be a response to difficulties with individuation, either being frustrated by overprotective parents or when the young person feels unready for adulthood, and ED behaviours are a way to remain childlike and maintain closeness to the parent [28, 29, 55]. These findings further suggest the entanglement of AN and concurrent developmental processes, such that for some people the AN is experienced as a response to thwarted individuation from parents.

Participants in the study shared their experiences of friends and family members’ emotional and behavioural responses to their AN. Participants described sensitivity to family and friends’ emotions, and the importance of them staying calm and regulating their emotions. This finding overlaps with previous study’s findings regarding the importance of emotional support and parents maintaining equanimity [43]. Participants in this study also shared a desire for flexible boundaries, finding both parental efforts to control their behaviour and increased permissiveness as unhelpful; rather, it was helpful to be given space to individuate from parents whilst parents continued to provide some limited oversight and feeling of containment. There is overlap here with the findings of the previous study in this area; Dimitropoulos and colleagues found that emerging adults valued practical support during treatment, but that it was important that this did not cross the line into control [43]. Participants in this study acknowledged that this freedom often resulted in worsening of ED behaviours in the short-term. Similarly, Dimitropoulos and colleagues reported that ambivalent feelings about recovery in the absence of parental control can provide opportunity for relapse [43]. However, this study adds the insight that, for some, worsening of the AN in the absence of parental monitoring may facilitate increased understanding of agency and independence, and ultimately facilitate movement towards recovery.

Finally, this study found that many participants had friends and family members with lived experience of ED. In some cases, this experience was perceived as detrimental to support efforts, whilst for others it was integral to good support. This is a novel finding in this emerging adult population. However, it is not surprising that people with EDs have family members with EDs; there is considerable evidence for the heritability of EDs [56]. Whilst a genetic component likely contributes to ED development, there is acknowledgment that direct and indirect parental behaviour such as encouragement of weight loss and modelling of body dissatisfaction and disordered eating can contribute to ED aetiology and maintenance [57, 58]. There is also existing evidence that friendships with others with EDs are often positively valued and can have a positive impact on recovery (59, 60,6).

This study has several strengths. It is a novel study which makes a unique contribution to the literature. Data have been analysed rigorously, using the steps outlined by the developers of IPA [47]. The study is reported according to the Consolidated Criteria for Reporting Qualitative Research Guidelines [48], promoting the comprehensiveness of the reporting, and allowing the quality and rigour of the work to be assessed.

A significant limitation of the study is the lack of diversity in terms of gender and ethnicity within the sample. Individuals of all genders and ethnicities are affected by EDs, yet this diversity is often not well represented in ED research [61]. It may be that individuals with certain identities may feel more unsure about responding to research advertisements and coming forward to share their experiences. It is also important to consider that given that participants were told that this was a study focused on support from family and friends there may be a lack of diversity with respect to experiences of support. It may be individuals who decided to participate felt they had particularly notable positive or negative experiences of support from family and / or friends self-selected to participate. It is also worth noting that this study did not collect data on when participants experienced AN onset (i.e., during adolescence or during EA). It was therefore not possible to explore the extent to which support might vary according to timing of illness onset.

There are also limitations of the IPA approach. The aim of IPA is to produce an in-depth examination of certain phenomena and does not aim to generate a theory that can be generalised to a whole population. However, given the lack of research on this topic, such an in-depth exploration provides a useful building block to develop further research. Future research might explore parents’ experiences of supporting emerging adults with EDs, and the perceived barriers and facilitators to support. It is important to seek to understand factors that may impact parents’ ability to adapt and meet the needs of emerging adult offspring, for instance physical and mental illness, marital conflict, unemployment and poverty, and cultural beliefs. Further research should also focus on the development and evaluation of novel interventions– or adaptations to existing interventions– arising from this increased understanding of support from family and friends during emerging adulthood. This study focused on understandings of social support once treatment had been completed, for reasons that have been outlined previously. However, it might also be interesting and worthwhile to explore emerging adults’ understandings of support whilst they are in treatment to understand how experiences vary or remain the same across the course of the illness.

The findings of this study suggest that clinicians may find benefit in helping emerging adults to develop their independence and supporting parents to adopt helpful emotional and behavioural postures that tackle the ED maintenance cycle. They might, for instance, encourage parents to focus on their own emotional regulation, and support parents to facilitate age-appropriate levels of independence and responsibility. Existing interventions may well provide useful frameworks for therapeutic work with this age-group; MANTRA, for instance, with its focus on facilitation of helpful carer responses and development of patient identity, may be particularly well-suited to emerging adult populations without the need for extensive adaptation [62, 63]. Other interventions such as family therapy may also have utility in this population, when adapted to allow for greater consideration of issues pertaining to developing independence [38].

## Data Availability

The datasets used and / or analysed during the current study are available from the corresponding author on reasonable request.

## Abbreviations

AN: Anorexia nervosa
BED: Binge eating disorder
BN: Bulimia nervosa
CAEDS: Child and adolescent eating disorder services
CORE-Q: Consolidated Criteria for Reporting Qualitative Research Guidelines
EDs: Eating disorders
FREED: First Episode Rapid Early Intervention for Eating Disorders
IPA: Interpretative phenomenological analysis
OSFED: Other specified feeding and eating disorders
UK: United Kingdom
YA-TBT-S: Young Adult Temperament-Based Treatment with Supports
